# Adult height and health-related quality of life in patients born small for gestational age treated with recombinant growth hormone

**DOI:** 10.1038/s41598-023-30281-z

**Published:** 2023-02-23

**Authors:** José Manuel Rial Rodrígez, Lourdes Ibañez Toda, Ignacio Díez López, Jordi Bosch Muñoz, Luis Salamanca Fresno, Eugenio Fernández Hernández, Antonio de Arriba Muñoz

**Affiliations:** 1 Pediatric Endocrinology Unit, Hospiten Rambla, Santa Cruz de Tenerife, Spain; 2 Pediatric Endocrinology Unit, Hospital Sant Joan de Déu, Barcelona, Spain; 3 Pediatric Endocrinology Unit, Hospital Universitario de Álava, Vitoria-Gasteiz, Spain; 4 Pediatric Endocrinology Unit, Arnau de Vilanova Hospital, Lleida, Spain; 5 Pediatric Endocrinology Unit, La Paz Hospital, Madrid, Spain; 6 Pediatric Endocrinology Unit, Hispalense Institute of Paediatrics Group, Sevilla, Spain; 7 Pediatric Endocrinology Unit, Miguel Servet Hospital, Av Isabel La Católica 1-3, 50009 Zaragoza, Spain

**Keywords:** Psychology, Health care, Medical research

## Abstract

Health related quality of life (HRQoL) is a relevant result when assessing the course of different pathologies and the efficacy of their treatments. HRQoL has been studied previously on adults born small for gestational age (SGA), both in the general population and in patients who had received recombinant human growth hormone (rhGH) treatment, with disparate results. Our study included 50 adults who had received rhGH treatment for the SGA indication in 4 Spanish hospitals. Data have been gathered retrospectively from their clinical records, current weight and height were measured, and patients have been asked to fill out SF-36 and QoLAGHDA quality of life forms, and the Graffar test to evaluate their socio-economical status. Patient’s adult height was – 1.2 ± 0.9 SD, lower than their target height of 1 ± 0.8 SD, but gaining 1.7 ± 1 SD from the beginning of the treatment. SF-36 test results showed lower scoring on Mental Health domains than on those related to Physical Health. No correlation was found between HRQoL results and final height, rhGH treatment duration or puberty. Correlation was indeed found between QoLAGHDA and several domains of SF-36, but QoLAGHDA detected fewer patients with low HRQoL than SF-36. Thus, it is concluded that SGA patient’s follow-up should include a HRQoL, neuro-cognitive and psychiatric assessment in their transition to adult age. Adult SGA patients without catch up growth have impaired HRQoL, especially in mental health domains.

## Introduction

Children born small for gestational age (SGA) are exposed to greater morbidity and mortality in the neonatal period; it has also been shown that low birth weight is associated with various pathologies during childhood and later in life. These pathologies include growth, pubertal and neuro-cognitive disorders, as well as metabolic syndrome and cardiovascular events in adults. For all these reasons, a specific follow-up of these patients is required^1,2^.

Health-related quality of life (HRQoL) reflects the perception of health as the physical, emotional and social well-being of an individual, and is a relevant outcome to assess the course of different pathologies and the efficacy of the treatments used. There are generic instruments (questionnaires) available that allow the study of HRQoL across different pathologies, and other tools designed for specific entities^3^. *The Quality of Life Assessment of Growth Hormone Deficiency in Adults Measure (QoL-AGDHA)* has been used to evaluate the effects of recombinant human growth hormone (rhGH) in adults with GH deficiency^4^. There are also specific tools to evaluate the HRQoL of children with short stature, which have been applied in studies carried out in subjects with SGA, idiopathic short stature, and on individuals with multiple pituitary deficits^5^.

In young adult SGA patients, generic tools have been used, such as the *Short Form 36 Health Survey (SF-36)*^6^*, the TNO-AZL Adults Quality of Life*^7^*, or the Child Health and Illness Profile: Adolescent Edition *(*CHIP-AE*)^8^, which have proved reliable and consistent in other pathologies.

The neuro-cognitive and learning problems of SGA children have been highlighted in several publications^9–11^. SGA subjects who experience catch-up growth seem to achieve better skills than those who do not^12^. There is some evidence that the intellectual performance of SGA without catch-up improves during treatment with rhGH^13,14^.

In general population, SGA adults achieve lower professional and economic goals than those born with adequate weight^15^, but there is no difference in their degree of life satisfaction or marital status^16^. HRQoL measured with the *SF-36* questionnaire in SGAs has shown similar^17^ or lower^18^ results than those obtained on individuals born with adequate weight.

In SGA patients without catch up growth, some studies report a better quality of life after rhGH treatment^19,20^ correlating with their height gain, showing similar HRQoL as the control population^21^ or improving their previous scores^22^.

In the present study, carried out on SGA patients without spontaneous catch up growth treated with rhGH according to the current guidelines of the European Medicines Agency (EMA)^23^, HRQoL was evaluated at the end of follow-up, comparing the results with the published references of the general population, and the correlation was analyzed between HRQoL results with auxological parameters and with the socioeconomic level.

## Patients and methods

### Patients and study design

This is a multicenter, observational and retrospective study including an adult SGA sample, followed in the Pediatric Endocrinology Units of four Spanish tertiary hospitals.

All patients had received treatment with rhGH under the SGA indication according to EMA guidelines. SGA was defined as a birth weight and/or birth length < − 2SD and treatment is indicated when the child have a height < − 2.5SD at 4 years old^23^. Patients with chronic diseases, hormonal deficits (pituitary, thyroid, pancreatic, adrenal or gonadal), severe postnatal sequelae, bone dysplasia and genetic-malformative syndromes, or obese [body mass index (BMI) > p97] were excluded. Patients with incomplete neonatal or parental height data and patients with poor adherence to rhGH treatment were also excluded.

A cohort of 169 patients who had been treated with rhGH under the SGA indication and who had reached final height were selected. We offered them to participate in the study, but only 50 wanted to take part.

Retrospective data (perinatal and family data, auxologic data at the start of treatment and during follow-up, registered side effects) were collected from medical records, and adult weight and height were measured during a face-to-face visit after the end of treatment, during which socioeconomic status was also estimated and quality of life was assessed.

### Anthropometric data and questionnaires

Anthropometric data were converted to standard deviations (SD) from the Spanish Collaborative Study of Growth 2008^24^. Response to treatment with rhGH was evaluated at 1 and 2 years, expressing height gain in ΔSD from initial height. Treatment efficacy was assessed by calculating the ΔSD of adult height compared to initial height, and its difference with the target or genetic height.

Socioeconomic level was estimated using the Graffar test, which arranges patients into different social classes (I–V) according to their profession, educational level, income sources, housing and neighborhood^25^. The scale that the family occupies in society is obtained based on the sum of these scores. Families with the highest strata (I and II) belong to the highest level of well-being, while families in relative poverty and extreme or critical poverty belong to the lowest strata (IV and V).

The *QoL-AGHDA and SF-36* questionnaires were used to assess HRQoL. The *QoL-AGHDA* is a simple, self-administered questionnaire containing 25 questions with Yes/No answers, which takes a few minutes to complete. To each affirmative answer one point is assigned. The points are added to give a single index ranging from 0 to 25, and a higher score indicates a worse HRQoL^4^.

*SF-36* questionnaire is an internationally accredited instrument, and has been validated in Spanish language^26^. It includes 36 items grouped into 8 domains: physical functioning (10 items), physical role (4 items), bodily pain (2 items), general health (5 items), vitality (4 items), social functioning (2 items), emotional role (3 items) and mental health (5 items). The scores obtained in each domain are transformed to a scale ranging from 0 (worst) to 100 (best). The 8 resulting scales are in turn grouped into two summary measures: Physical Health and Mental Health, which are the result of adding the previous scores corrected by a coefficient^27^. The summary scores are compared with the percentile values of a general population sample. An additional item, Evolution of Health (EV), is not included in the summary measures and is separately assessed, reflecting the changes in health perception since the previous year, from much better (= 1) to much worse (= 5)^28^. All surveys were reviewed and analyzed by the same observer.

### Statistical study

A normality study was initially performed to apply parametric or non parametric tests and, subsequently, depending on the variables analyzed, appropriate tests were applied, considering statistical significance when p < 0.05 with a confidence interval (CI) of 95%. The statistical treatment was performed with the IBM SPPS Statistics 22 program for Windows.

### Ethics approval

Prior to the study, approval was obtained from the different Research Ethics Committees of the four participating hospitals (Hospital Universitarui Arnau de Vilanova Research Ethic Committee, protocol v.2018, HIP/CI v.7/2019). Informed consent was obtained from all the patients to access data in their medical records and to carry out the face-to-face visit in adulthood.

## Results

A sample of 50 patients were studied, 37 females (74%). Table 1 shows the data at birth and parent height. Only one patient had been born after multiple gestation, and seven were preterm (gestational age 30–36 weeks). Ninety percent suffered no perinatal problems; the remaining 10% reported mild anemia, jaundice or respiratory distress.

**Table 1:** Data at birth and parental height in patients born small for gestational age without postnatal catch-up growth (n = 50).

	Mean ± SD
Gestational weeks	38.7 ± 2.6
Birth weight (SD)	− 2.2 ± 1.0
Birth length (SD)	− 2.8 ± 1.2
Maternal height (SD)	− 0.9 ± 1.2
Paternal height (SD)	− 0.7 ± 1.0
Target height (SD)	− 1.0 ± 0.9

*SD* standard deviation.

The rhGH treatment started at an age of 7.6 ± 3.0 years, and its duration was 7.1 ± 2.8 years; mean cumulated height gain in the first year was 0.8 ± 0.8 SD and 1.2 ± 1.1 SD in the second year. Adult height was − 1.2 ± 0.9 SD, slightly lower than the target height of − 1 ± 0.9 SD (Table 2). No side effects related to the treatment were reported in any patient. IGF-1 values remained in the normal range at all times. All patients completed their treatment to final height and there were no premature discontinuations.

**Table 2:** Change in anthropometric parameters during recombinant human growth hormone (rhGH) treatment and adult height in subjects born small for gestational age without catch-up growth (n = 50).

	rhGH start	1st year	2nd year	Adult
Age (years)	7.6 ± 3	8.6 ± 3.0	9.2 ± 3.0	21.2 ± 2.8
Weight (SD)	− 1.9 ± 0.7	− 1.2 ± 0.7	− 0.8 ± 0.8	− 0.9 ± 1.1
Height (SD)	− 2.9 ± 0.6	− 2.0 ± 0.8	− 1.5 ± 1.0	− 1.2 ± 0.9
Height gain (SD)^a^		0.8 ± 0.8	1.2 ± 1.1	1.7 ± 1.0
BMI (SD)	− 0.7 ± 0.6	− 0.7 ± 0.7	− 0.7 ± 0.6	− 0.6 ± 0.9
HV (SD)	− 1.3 ± 1.3	3.0 ± 2.7	2.3 ± 1.5	
HV increment (SD)^a^		4.3 ± 2.9	3.5 ± 2.1	
rhGH dose (mcg/kg/day)	38.6 ± 14.4	38.7 ± 13.6	38.7 ± 14.6	

*BMI* body mass index, *HV* height velocity, *SD* standard deviation.

Values are expressed as mean ± SD. ^a^From the start of rhGH treatment.

Thirty-eight patients (76%) were prepubertal at treatment start, and 12 were in Tanner stage II–III. During the treatment puberty started at 12.1 ± 1.2 years in boys and at 10.6 ± 1.3 years in girls. In 4 girls with low predicted adult height (predicted adult height < 150 cm with a difference of more than 10 cm with respect to their target height), treatment was associated with GnRH analogues (GnRHa), at 8.3 ± 0.7 years old, 125.1 ± 2.3 cm and a bone age of 11 ± 0.3 years, reaching a final height of − 1.4 ± 0.7 SD. The remaining women reached a final height of − 1.3 ± 0.8 SD (p = 0.4). The age at menarche was 14.1 ± 1.0 years (rhGH + GnRHa) vs 13.0 ± 1.2 years (rhGH) (p = 0.15). Between both groups, no differences were observed in the age at the end of treatment, in BMI, or in the results of the quality of life questionnaires.

At the age of 21.2 ± 2.8 years and 6.5 ± 2.8 years after the end of treatment with rhGH, the patients were called again to measure their height and weight and to fill out the questionnaires. According to the Graffar test, 23% of the sample belonged to group I (highest class), 31% to group II, 35% to group III, and 11% to group IV.

**Figure 1:**
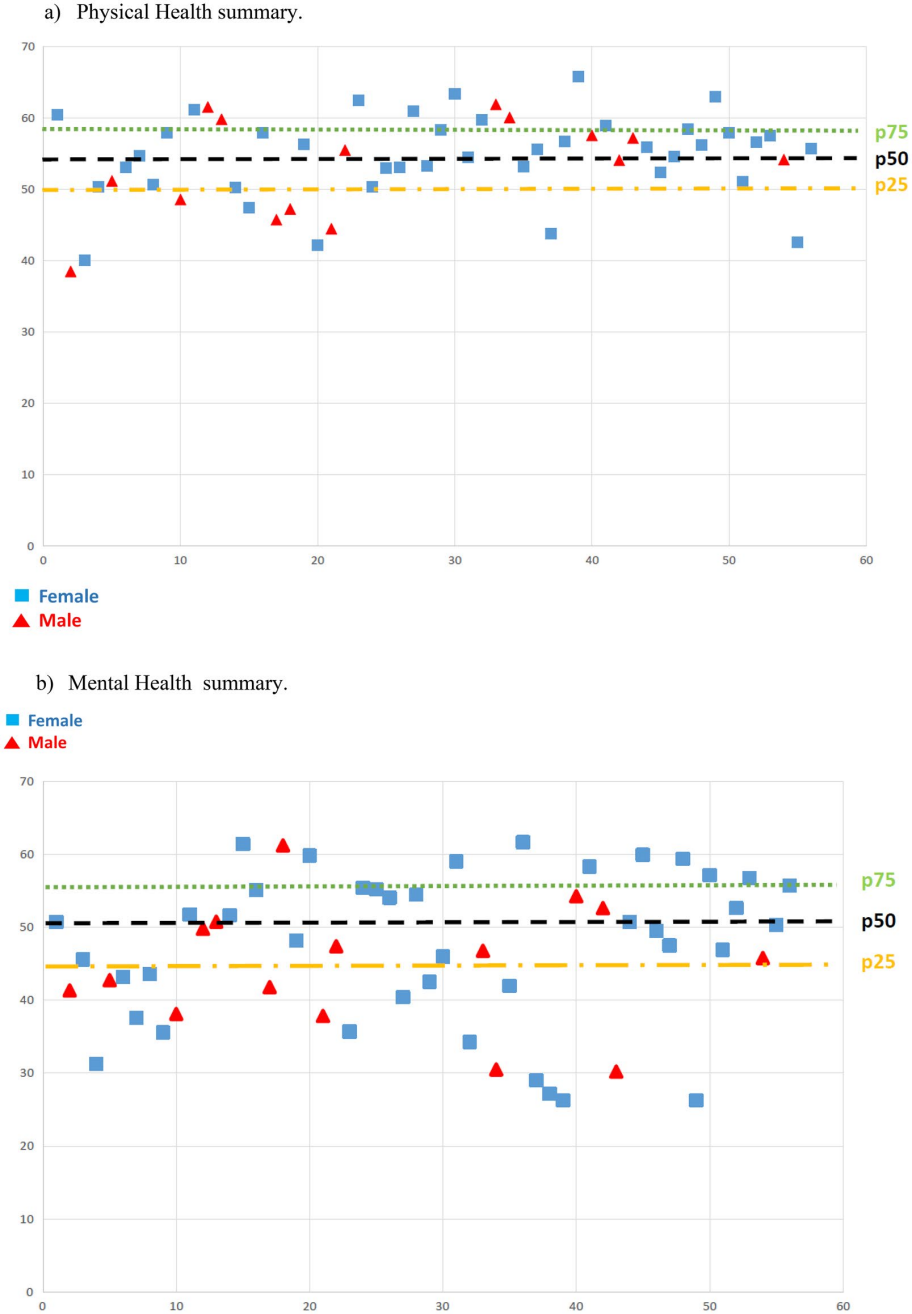
(**a**) Physical health summary. (**b**) Mental health summary.

In the *SF-36* questionnaire the physical functioning and physical role domains had the best scores, and the vitality and mental health domains the worst. Concerning the summary measures, better results were found for Physical Health than for Mental Health, laying both mean values below the population references (Table 3). Below the 25th percentile were 28% of patients for physical health and 38% for mental health. Males scored lower, 53% below the mean in physical health and 77% in mental health summary measures (Fig. 1).

**Table 3:** Overall results of the quality of life questionnaires.

SF 36			QoLAGDHA correlation
	Mean	SD	
Evolution of health	2.67	0.88	
Physical functioning	96.10	8.65	− 0.41
Physical role	92.50	20.36	− 0.34
Emotional role	78.76	31.42	− 0.62
Social functioning	77.65	23.23	− 0.27
Bodily pain	75.86	24.92	
Vitality	66.90	18.32	− 0.42
Mental health	70.08	17.58	− 0.42
General health	73.04	20.93	
SF 36 mean	78.90	13.01	− 0.62
Summary physical health	54.34	6.19	
Summary mental health	46.74	10.35	− 0.42
QoLAGHDA	4.8	4.15	1

All showed correlations p < 0.05.

The age of the patients when questionnaires were filled out correlated positively with physical health (r = 0.280, p = 0.04), while physical role was positively associated with target height (r = 0.371, p = 0.005) and the age at rhGH start (r = 0.31 p = 0.019), and negatively correlated with the difference adult height SD—target height SD (r = − 0.27, p = 0.044), and adult weight (r = − 0.54, p = 0.021).

The mean value of the declared evolution of health status was 2.7 ± 0.9, and the mode (most frequent value) was 3, indicating the absence of changes over one year. Health status evolution was positively correlated with the age at treatment start (r = − 0.29 p = 0.046) and negatively correlated with its duration (r = − 0.31 p = 0.029), thus scoring worse when treatment was started later.

In *QoL-AGHDA* questionnaire, only 25% of patients scored above the mean (indicating worse quality of life), and only 14% exceeded 1 SD, showing a lower sensitivity than the *SF-36.*

The *Qol-AGHDA* score correlated with the target height SD—final height SD difference (r = − 0.28, p = 0.04) and with the *SF-36* mental health scores (r = − 0.42, p = 0.04), physical functioning (r = − 0.41 p = 0.04), physical role (r = − 0.34 p = 0.02), emotional role (r = − 0.62 p = 0.00), social function (r = − 0.27 p = 0.01), vitality (r = − 0.42 p = 0.007), mean SF-36 (r = − 0.62, p = 0.00) and the mental health summary (r = − 0.42 p = 0.00). There was no significant correlation with general health (r = 0.25 p = 0.86), bodily pain and physical health summary (r = 0.11 p = 0.46) (Table 3).

GnRHa treatment had no impact on the quality of life questionnaires results.

No correlation of HRQoL score with socioeconomic status was found.

## Discussion

The mean of 1.7 SD height gained by our patients from the start of rhGH is similar to that achieved in other series^29^, and final heights were slightly lower than target heights.

Puberty onset was in the range of the reference population^30^ and age at menarche, excluding the 4 patients treated with GnRHa, was about one year later compared to other SGA cohorts^31^. Treatment with rhGH does not seem to affect puberty timing in SGA^32^ although spontaneous catchup growth in SGA may be associated with pubertal advancement^33^. GnRHa treatment did not affect the HRQoL questionnaires results, as described in other studies^7,22^.

The HRQoL has been previously studied in SGA adults with heterogeneous results. For example, in an Irish cohort of 111 SGA adults born in a single maternity hospital in Belfast, no differences in *SF-36* scores were found compared to normal birth weight controls^17^. Conversely, in another cohort of 55 adult PEG from Norway, a HRQoL decrease in the mental, emotional and social domains of *SF-36* was observed, linked to an increase in psychiatric pathologies^18^.

SGA population without catch up has been previously investigated using generic tools (*EQ-5D*^20^;* SF-36*^6^;* TAAQOL*^7^), as well as specific ones for short stature (*QoLISSY*^21^). There is currently no specific instrument available for SGA adults without catch up growth. We have used the *SF-36* as a generic instrument, and the QoL-AGHDA, designed for adults with growth hormone deficiency, to establish its correlation with *SF-36* and assess its usefulness in SGA treated to final height.

In contrast to Sommer's^6^, our multicenter study shows that SGA who complete treatment with rhGH score below the standard mean on the *SF-36*, being below the 25th percentile, (which indicates a moderate difference with respect to the population) 28% in physical health and 38% in mental health summary measures. Other authors have found lower scores in mental health, emotional role and social function domains^18^ and a higher prevalence of psychiatric pathologies in SGA. This pathology can worsen from adolescence to adulthood^34^. However in our cohort, better *SF-36* mental health values were associated with an older age at the time the questionnaire was filled, and with the years elapsed since the end of treatment, which could be interpreted as an increasing resilience in problem facing with age. Worse scores in male patients, together with their lower proportion in the sample, suggest that, as participation in the study was voluntary, it could lead to a biased inclusion of those with a poorer perception of their health.

We found no correlation of HRQoL with the main targets of rhGH treatment, such as adult height SD, or the difference between adult height SD and genetic height SD. Only the physical role domain correlates with this difference. Adult weight correlates negatively with mean *SF-36* score and with physical role. (Table 3) Neither physical role nor health evolution have a significant weight in the calculation of the mental health summary measure, which is the most affected parameter. The low correlation between adult height and HRQoL in our series is not surprising, since most of the patients reached a height within normal limits. On the other hand, this discordance has already been described both in the general population and in patients with short stature of different etiologies^35,36^.

The effects of rhGH in deficient subjects go beyond the increase in height, and can act directly on the perception of health and well-being^37^. In our patients, the time elapsed from the end of treatment rules out any possible effect of the treatment on HRQoL outcomes. Nor could it be attributed to treatment discontinuation, since it is known that in adolescents with growth hormone deficiency, ending treatment upon reaching final height had no effect on their HRQoL^38^.

Neither did we detect significant correlations between HRQOL and socioeconomic level, a possible confounding factor.

Regarding the *QoL-AGHDA*, no significant correlations were detected with aspects related to rhGH treatment such as age at start, dose, or adult height, excluding the difference between target height and final height. The *QoL-AGHDA* results present a negative correlation with the domains related to mental health, and also with the physical function and physical role domains of the *SF-36* questionnaire, so it can be concluded that both explore similar features. However, the *QoL-AGHDA*, being easy to complete and assess, is specifically designed for adults with growth hormone deficiency, and identifies only 14% of SGA patients in our sample with low HRQoL, showing lower sensitivity than the *SF-36*.

However, the *SF-36* does not investigate important aspects such as sleep, cognitive function, or sexual activity, so the addition of a neuro-cognitive and psychiatric assessment would provide a more complete picture of the HRQoL of SGA patients at the end of follow-up, in their transition to adulthood.

As the inclusion for this study was voluntary, this can influence the results, but we don’t know if this could be in a positive or negative way; maybe patients with problems participated and patients without problems not, or the other way around. In addition, although the sample size is not very large, studies such as this one are important to carry out in order to detect the quality of life problems that patients may present. Looking to the future, it would be very interesting to carry out prospective studies to evaluate the quality of life of patients at the beginning of treatment and then annually to see how it evolves.

## Conclusions

1. Adult SGA patients without catch up growth have impaired HRQoL, especially in mental health domains.
2. Such impairment is not related to height attained or to other parameters related to treatment with rhGH.
3. The selective involvement of mental health domains suggests that these patients should be investigated for neuro-cognitive and psychiatric issues that may affect HRQoL in adulthood.
4. The *QoL-AGHDA* questionnaire shows significant correlation with the physical and mental domains of the *SF-36,* although it has less sensitivity for detecting a decline in HRQoL in our patients.

## Data Availability

Data are held by the authors and can be accessed at any time by any researcher. If someone wants to request the data from this study, they can contact Antonio de Arriba Muñoz (adearriba@salud.aragon.es).
